# Testing Vision Is Not Testing For Vision

**DOI:** 10.1167/tvst.9.13.32

**Published:** 2020-12-18

**Authors:** Eli Peli

**Affiliations:** 1 Schepens Eye Research Institute of Mass Eye & Ear, Department of Ophthalmology, Harvard Medical School, Boston, MA, USA

**Keywords:** visual perception, vision restoration, prosthetic vision, sensory substitution, spatial perception, optogenetic, stem cell, gene therapy

## Abstract

Visual prostheses aim to restore, at least to some extent, vision that leads to the type of perception available for sighted patients. Their effectiveness is almost always evaluated using clinical tests of vision. Clinical vision tests are designed to measure the limits of parameters of a functioning visual system. I argue here that these tests are rarely suited to determine the ability of prosthetic devices and other therapies to restore vision. This paper describes and explains many limitations of these evaluations.

Prosthetic vision testing often makes use of multiple-alternative forced-choice (MAFC) procedures. Although these paradigms are suitable for many studies, they are frequently problematic in vision restoration evaluation. Two main types of problems are identified: (1) where nuisance variables provide spurious cues that can be learned in repeated training, which is common in prosthetic vision, and thus defeat the purpose of the test; and (2) even though a test is properly designed and performed, it may not actually measure what the researchers believe, and thus the interpretation of results is wrong. Examples for both types of problems are presented. Additional problems arise from confounding factors in the administration of tests are pointed as limitations of current device evaluation. For example, head tracing of magnified objects enlarged to compensate for the system's low resolution, in distinction from the scanning head (camera) movements with which users of prosthetic devices expand the limited field of view. Because of these problems, the ability to perform satisfactorily on the clinical tests is necessary but insufficient to prove vision restoration, therefore, additional tests are needed. I propose some directions to pursue in such testing.

**Translational Relevance:**

Numerous prosthetic devices are being developed and introduced to the market. Proving the utility of these devices is crucial for regulatory and even for post market acceptance, which so far has largely failed, in my opinion. Potential reasons for the failures despite success in regulatory testing and directions for designing improved testing are provided. It is hoped that improved testing will guide improved designs of future prosthetic systems and other vision restoration approaches.

## Introduction

Vision restoration for the blind attempts to restore, at least to some extent, the visual perception of the type we know to be available for fully or partially sighted individuals. I define “vision” here, as a sensory experience that results in perception with spatial-temporal characteristics typical of the natural visual system, although it may be of lower resolution and lower temporal bandwidth. Such vision should support the ability to convert a 2D (sensory) image back to a 3D perception of the scene from which it was derived (the inverse problem^1^). This may be achieved using monocular cues (e.g. perspective, occlusion, relative size, and motion parallax). Such vision will exhibit Gestalt properties,^2^ such as good continuation, closure (reification), common fate, common region, element connectedness, proximity, etc. Vision enables us to recognize objects from novel viewpoints and to identify novel objects after seeing examples from the same category (generalization). Vision is used to control our self-actions, and discounts self-motion in the environment, enabling us to perceive a stable world as we move through it (even in virtual motion) and distinguish other motions within the sensory input. Such vision will also enable figure-ground segregation. Restored vision should exhibit as many of these properties as possible. It is necessary to demonstrate that prosthetic devices or other vision restoration modalities, such as gene therapy, optogenetics, or stem cell therapy, provide these capabilities.

I explicitly distinguish visual prostheses designed to restore vision from visual aids that are not designed or required to provide vision or visual experience. For example, text-to-speech systems provide reading capabilities but do not restore vision. The long cane, an amazingly effective mobility aid, enables blind people to commute without restoring vision. Visual sensory substitution devices (SSDs) using non-vision input (i.e. hearing^3^ and tactile^4^) may be considered to be visual prostheses. I reserve the term “visual SSD” for systems that are intended to provide vision through another sensory modality. This is distinguished from cross modal aids (e.g. Braille or text to speech applications). In an analogue way, I consider the cochlear implant to be an auditory restoration device, whereas sign language and captions for the hearing impaired to be merely auditory aids. Testing a visual prosthesis should first and foremost determine if the prosthesis provides vision. Secondary testing can then evaluate the performance parameters of the system. Unfortunately, the testing is frequently limited to the secondary parameter's evaluation skipping the crucial proof of visual perception.

It is not known if SSDs, such as tactile stimulation of the skin,^5^ electrical stimulation of the tongue (for example, the BrainPort^4^; Wicab, Inc.; Middleton, WI), or visual-to-auditory sensory substitution devices^6^^–^^9^ lead to visual perception. All these SSD systems lead to a sensory response, but that is not necessarily vision as it is defined above. It is established that electrical retinal stimulation systems like the Argus II^10^ (Second Sight, Sylmar, CA), the Alpha IMS^11^ (Retina Implant AG, Reutlingen, Germany), the IRIS II (Pixium Vision, Paris, France), or the PRIMA^12^ (Pixium Vision) result in visual sensory responses (phosphenes). This, however, does not mean that these retinal prosthetics restore vision, as defined here. In fact, 16 patients implanted with either the Argus II or the Iris II system described their perception of the experience “as fundamentally, qualitatively different than natural vision,”^13^ and those perceptions did not change over time resulting in the eventual abandonment of the use of the devices.^13^

One could argue that if a visual aid provides any discrimination of light from dark, it is a vision restoration system (light perception). I would concede that such a definition may be acceptable in some general sense, but such a general definition is neither useful nor helpful in the context of visual prosthesis for the blind and the evaluation of vision restoration systems or treatments, even if that minimal capability enables the user to perform better on some visual tasks. A single light sensor system may be of some limited use, but it needs not be implanted and can be provided at extremely low cost and risks. However, no one considers such a simple visual aid to provide vision restoration. Such performance does not justify the cost and risk of implanted prosthetics or other invasive vision restoration systems. I will argue here that most current evaluation methods of prosthetic systems that use standard clinical or psychophysical vision test paradigms do not demonstrate that the systems actually restore vision, despite the ability of the users to perform above chance on the clinical tests. In fact, some testing explicitly showed that the performance is essentially equivalent to a single sensor system.^14^^,^^15^

Standard clinical vision testing procedures are not designed to test the ability of prosthetic devices to restore vision, and thus they are inappropriate measurement tools. Yet, there is an effort to develop a consensus about the use of these techniques in evaluating prosthetics.^16^ The vision tests used in clinics and psychophysics laboratories (i.e. perimetry, color vision testing, acuity, contrast sensitivity, and orientation discrimination tests) are designed to measure the limits of a working, functioning visual system that meets our perceptual needs. They are not designed to determine if the visual system sees. The fact that the patients (normally or partially sighted) see is taken for granted in the design and application of these tests.

The ability to perform well on the clinical tests is necessary but insufficient to prove vision restoration. For example, it is not possible to interpret perspective without being able to distinguish retinal line orientation. Yet, being able to discriminate line orientation on a multiple-alternative forced-choice (MAFC) test is not an indication of the ability to interpret perspective. Likewise, it is not possible to parse depth using self-motion (motion parallax) without being able to discriminate speed differences or direction. Yet, above chance performance on a motion discrimination task does not mean one can use motion parallax to determine the 3D structure of a scene. Apparently, such capabilities do not develop over time with the use of prosthetic vision systems.^13^ Importantly, some MAFC tests used to determine motion detection or discrimination fail in demonstrating such capabilities because they can be passed without perceiving any motion.^14^

There are two types of problems with the use of clinical tests in evaluating prosthetic vision; (1) With the extensive training needed and applied with prosthetic vision devices, the users may learn to defeat the MAFC test using unintended secondary, possibly non-visual cues. This is rarely a problem in a single clinical encounter or even in a properly designed laboratory procedure. (2) If a test is not designed to explicitly test for the presence of vision, passing it or demonstrating a certain performance level may be wrongly interpreted as an indication of vision. This problem is not specific to MAFC, it is just the paradigm used most often in the evaluation of prosthetic vision systems. MAFC testing can be used to determine the resolution limits of visual prostheses or other specific parameters. This is an appropriate use and interpretation of the testing paradigm. By itself, however, it does not tell us much about the perception of the users. Frequently, the first problem may defeat even the ability to accurately determine simple parameters, such as thresholds.^14^

## Example – Restoring Normal Color Vision?

We rarely test to determine the existence of vision, as it is usually not necessary. Color vision is a case where testing is explicitly done to determine if the patient has normal (trichromatic) color vision. In addition to testing for normal trichromacy, the color vision test also tests for the type (i.e. protanope or deuteranope) and level of the anomaly, if present. If a patient who is known to be a dichromat passes a color vision test using a device, does that mean that the device restored normal trichromatic vision? The answer is clearly, no. Similarly, passing a vision test with a prosthetic vision system does not prove that the type of vision assumed by the test actually exists. To further illustrate this distinction, let us consider a few examples related to color vision testing.

Pseudo Isochromatic plates, such as the Ishihara Color Vision Test (Keeler Instruments, Malvern, PA), were developed to distinguish people with common color deficiencies from people with normal color vision. A dichromat wearing tinted glasses, such as the Enchroma Glasses (Berkeley, CA), may pass the test but does not have his/her color vision restored to normal trichromatic vision. In fact, the user's color vision is neither restored nor improved. A dichromat wearing a colored filter is still a dichromat and would fail on another pseudo isochromatic set of plates designed for use with the filter. Trichromatic normally sighted observers will pass both tests. The Ishihara and other similar tests are designed to be administered under very strict conditions, including the spectrum of the light cast on the plate. Any violation of these conditions, such as the use of tinted lenses, invalidates the test results and conclusions. The color filter may be considered a visual aid, helping a person to pass the test but it does not restore normal color vision. Under some circumstances, it may also help the patient to perform specific daily tasks.

A common daily activity that may require normal color vision is the discrimination of red from green on a traffic light. This task is particularly difficult for patients with red-green deficiency and poor acuity, even within the acuity level required for a driving license. A narrow red filter strip applied to the top of the spectacle lens enables the dichromat, slightly visually impaired, driver to easily discriminate the red traffic light from the green.^17^ The driver briefly tilts his head down to spot the light through the red filter. If the traffic light is red, it will shine brighter through the filter; if it is green it will become very dim or completely invisible. This is a real-life example of a color vision test. Yet, even 100% correct performance on this task does not indicate restoration of normal color vision. This device is a color vision aid, not a color vision restoration system.

Because training and testing using MAFC paradigms is a major approach to evaluating visual prostheses, consider another example of that in the color domain. A recent paper^18^ examined the ability of dichromats to perform on the Farnsworth D15 clinical color vision test following practice. Subjects were permitted to train on the Farnsworth D15 at home for as long as they wished. Of the 10 color-deficient subjects, 5 subjects achieved perfect Farnsworth D15 arrangements on their return testing visit. The other five subjects also significantly improved their performance. Reported practice time (mean of only 70 minutes) did not differ between those that achieved perfect Farnsworth D15 performance and those that did not. These subjects had no treatment and they certainly remained dichromats, yet half of them were able to perform perfectly on the Farnsworth D15. The Farnsworth D15 is a MAFC test and this result illustrates that with training people can defeat such tests without actually gaining the visual property (trichromatic color vision) the test is designed to check for. Several of the subjects “casually reported that grouping caps based on brightness assisted them in making some of the determinations,”^18^ indicating that they used a luminance cue to defeat the hue-based test. Thus, this is an example of the first problem with MAFC stated above. These results should serve as a caution against the use of the Farnsworth D-15 or Farnsworth-Munsell 100 hue tests in evaluating vision restoration, as was recently done in a gene therapy study.^19^

In the context of color vision restoration, it is worth considering the amazing achievement of gene therapy that introduced a third (red, L) photo-pigment into some (green, M) cone cells of dichromatic monkeys.^20^ Before treatment, the monkeys could detect a red disc but not a green disc on a grey background. With the new pigments in their retina, they were able to detect both color discs on the grey background. This may appear to be restoration of trichromatic color vision, but this test does not prove that. As argued by Cornelissen and Brenner,^21^ this test does not show that the monkeys could distinguish the red disc from the green disc, only that they can detect both discs. The ability to distinguish the red disc from the green disc was not tested. The ability to distinguish colors should be considered a minimum requirement for demonstrating restoration of trichromatic color vision. Such ability requires more than the presence of a third pigment in the retina, which was indeed proven by the original gene therapy study.

Color vision also requires the establishment of appropriate connections from the modified cones in the retina to the brain to establish trichromatic vision that was not available prior to the treatment. Despite the fact that the cones with the new pigments were separately connected to the brain, no evidence was presented that they were interpreted differently than they were before the red pigment was added. Thus, they continued to be interpreted as if they were green cones even though they have improved long-wave sensitivity. Thus, the treatment changed the quantal catch of the photoreceptor, but the quantal catch is not correlated with color appearance, as classically shown by Land.^22^ A change in color appearance would be a measure of change in color vision. A test for that critical question can be developed and should be applied. Cornelissen and Brenner^21^ proposed such a test (including training the monkey to distinguish red from green discs). They then demonstrated with simulations that simply replacing the pigment in some cones with the new pigment will not provide that ability. I would argue that even performing the task proposed by Cornelissen and Brenner (distinguishing the red from the green disc) may not be sufficient to prove restoration of trichromatic vision. Having the pigment inserted only in a portion of the retina^20^ may enable the monkeys to perform the task by comparing the view of the test targets between the modified and unmodified retinal areas (different eccentricities). Noting the difference between these two views is similar to the use of a partial color (red) filter on glasses, which enable a color-deficient driver to distinguish the red from the green traffic light. Thus, similar performance may be achieved with a simple partial filter, which does not restore trichromatic color vision. Such performance by a monkey would be evaluated using a two-alternative force choice (2AFC) paradigm, which is susceptible to the artefactual eccentricity cue. It is important to note that even successful performance on a properly designed test that avoids such artefacts and correctly proves performance that is only possible with trichromatic color vision does not say much about the perception of color by the monkey with the modified cones.

## MAFC Testing of Prosthetic Vision Function

Bach et al.^23^ developed a Basic Assessment of Light and Motion system to test patients with ultra-low vision using paradigms sometimes called 2AFC or four-alternative forced-choice (4AFC).^24^ They tested basic functions: light perception with an unstructured large-field; single versus double flash discrimination; localization of light in one of four possible directions; and motion in one of four directions. These were administered to people with severely impaired vision and were found to be effective in discriminating people with no light perception from those with light perception and, in some cases, from people with only “hand motion” vision. The use of MAFC in this case is appropriate as it is applied to natural (impaired) vision and attempts to measure threshold performance. The utility of the system for testing prosthetic vision was deemed by the authors^23^ to be useful only for extremely low vision, poorer than hand motion. One would hope that prosthetic vision provides better quality of vision than that, so this technique is unlikely to be of much use except perhaps to certify candidates for prosthetic or other treatment prior to inclusion in a study. In any case, it does not provide any resistance to artefacts, especially with repeated training, and performance on it will not prove the restoration of vision we are seeking to document.

Although it may appear self-evident that retinal stimulation devices provide vision, this cannot be regarded as established and proven, because current evidence is far from conclusive.^13^ On the other end, it is somewhat hard to conceive that SSDs, such as visual-to-auditory devices,^3^^,^^6^^–^^8^^,^^25^^,^^26^ provide visual perception. They certainly provide a sensory experience and have been demonstrated to enable performance on “visual” tasks. However, the existence of visual perception with these devices needs to be proven as part of their evaluation. Perhaps because it is harder to believe that vision is achievable with auditory SSD systems, attempts to demonstrate visual abilities have been reported.^6^^,^^8^ Between these two extremes are the tactile and electrotactile SSDs.^5^ Tactile SSD systems do provide a spatio–temporal stimulation that resembles that of vision but through sensory organs that may not convey visual perception. Evaluation of all these devices has relied primarily on MAFC testing, as commonly applied in clinical or psychophysical studies. I argue that these paradigms are vulnerable to artefacts by which subjects may be able to perform the test at above chance levels in the absence of vision of the type defined above. Hallum and Dakin^14^ pointed out that, although motion discrimination may appear to indicate some vision restoration, in fact, when tested with MAFC subjects may be able to defeat the test by noting the time of arrival of a moving bar at one end of the display or another. They argue that such performance can be achieved with a wide single sensor, implanted or otherwise. Note also that the electrical stimulation does not necessarily have to be visual to provide the timing information. The problem with these outcome measures is that it is erroneous to interpret such results as indicative of vision restoration.

**Figure 1.**
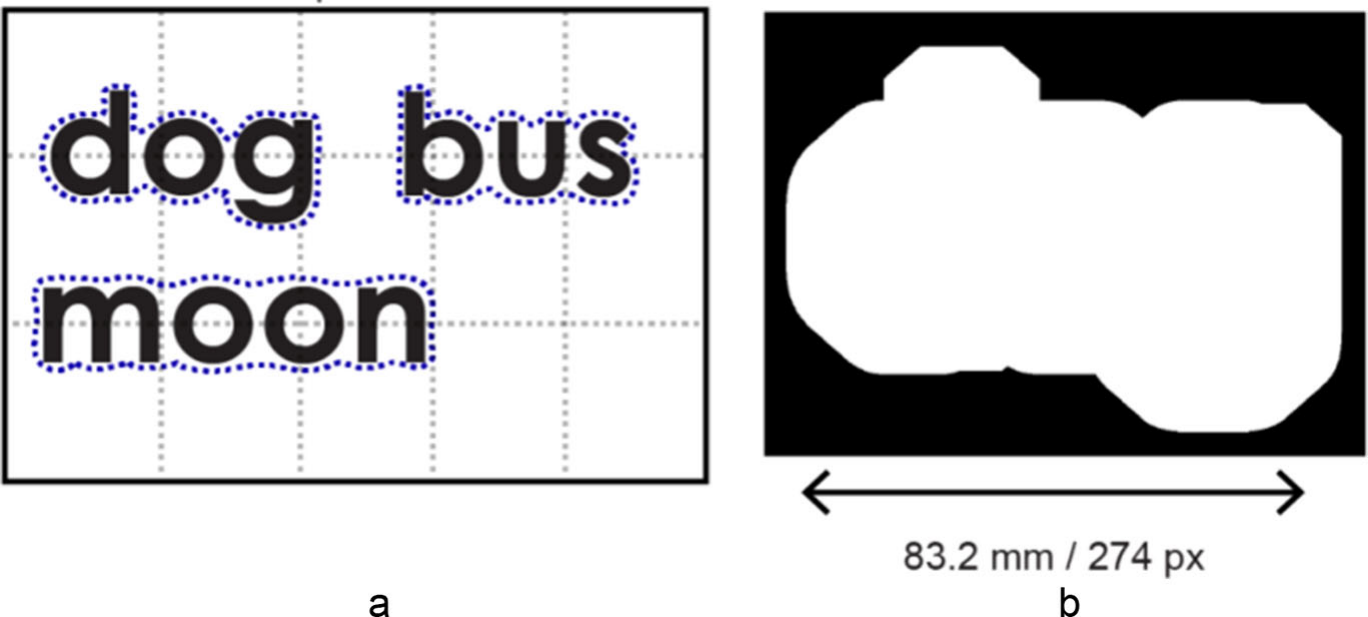
Illustrations of the low-resolution visual representation of word images used in the Han et al.^27^ study. (**a**) Three of the 10 words showing the crude outline in blue dots. Note the difference and possible cues in the position of ascending and descending letters (**b**). The actual image of the word “dog” as it was presented on the computer screen in the study. Adapted from Han et al.^27^

In a clinical trial of the BrainPort V100 tongue stimulation device,^4^ most blind subjects fell short of “recognizing” 6 of 10 words they were repeatedly trained on. The goal of 6 of 10 was set halfway between 10% chance level for correctly responding to 10 words and 100% correct performance. There were, however, three short (3 letters) words, 4 medium length (4 letters) words, and 3 long (5 letters) words. Considering this variation in length would increase the chance of correct guessing to 25% to 30%, which would require a threshold of correctly naming 7 of 10. The word length could be determined in testing by just scanning the head mounted camera across the word image, noting the required angle of motor scan without recognition of any additional spatial details. Additionally, the words in each length class varied in the use of ascending and descending letters at the beginning and end of the words (Fig. 1A), enabling near perfect performance with these specific 10 words based on the crude outline of the word shape in regard to the position of ascenders and descenders without being able to read any letters. Failing the test under these conditions proves that the device did not provide text reading vision in that study (failing the necessary condition).^4^ Passing the test, however, would not have proven the opposite because of these confounding factors of letters in the MAFC paradigm used.

**Figure 2.**
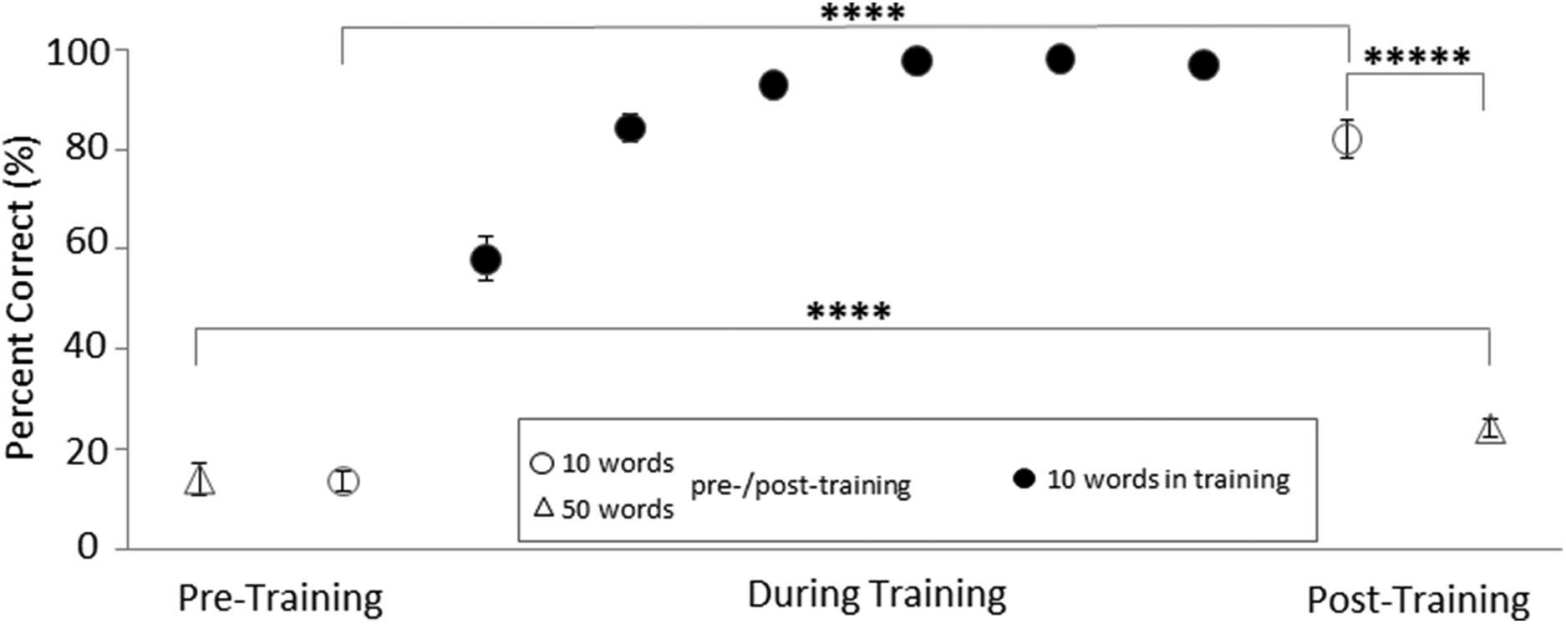
Improvement in correct recognition of the word images with training. A dramatic improvement in the 10 trained words compared to the 50 untrained words is apparent. The improvement during training is rapid and reaches about 100% quickly. The performance in the post-training test is slightly reduced due to the mix with the 50 untrained words in that test. Adapted from Han et al.^27^ ****: *P* < 0.0001, *****: *P* < 0.00001.

To examine this assertion, we have replicated the study design with normally sighted subjects.^27^ The 10 word images were processed to remove almost all details except for the word length and the position of the ascenders and descenders (see Fig. 1) and presented on a visual display. The subjects were trained with these 10 degraded word images the way they were trained in the prior BrainPort studies^4^ (for a total of 150 trials), using their normally functioning visual system. We also selected 50 additional commonly used words, matching the proportion of word lengths in the set of 10 trained words, and matching the proportion of words with ascenders and descenders. The 50 words were processed in the same way as the 10 trained words. The subjects were not trained on these 50 words. They were tested on the complete set of 60 words before and after the training on the 10 words (Fig. 2). At the end of the training, the subjects correctly named the 10 words presented on 97.5% of the trials, and all achieved the goal of 7 out of 10 correct. When the 10 trained words were tested mixed with the 50 untrained words the performance on the 10 words increased from 13.5% accuracy before training to 82% post training. In comparison, the increase in accuracy for the 50 untrained words in the mix was very small, from 14% to 24.1%. None of the subjects achieved the 50% threshold set as a goal for the 50 words. The testing paradigm and response format called the subjects attention explicitly to the word length and implicitly to the presence of the ascenders and descenders. Accurate determination of these features in the 50 words improved post training. These results show that even if a MAFC task is performed successfully after extensive training with the same targets, there is no guarantee of transfer to other, highly related tasks. Further, this was shown in a case where a normally functioning vision system was used. The results with untrained word images would be expected to be much poorer with the use of the tongue stimulation, which is not a vision system.

Normally sighted readers also use word length to improve their reading speed, but they use the word length cue against the full selections of words in the language. In Han et al.'s experiment, only 10 words were used. This is an example where the MAFC characteristics (limited number of response options) come into play in supporting the use of such spurious cues. They have shown that just increasing the selection to 60 words dramatically decreased the utility of this cue.

Caspi and Zivotofsky^15^ found that normally sighted subjects performed better on a MAFC Landolt C acuity test than should be expected based on the angular distance between a pair of electrodes in a simulated retinal implant array. The subjects used natural normal vision to interpret the displayed phosphene, which cannot represent abilities obtained with an implant-generated phosphene. Yet, their results demonstrate that a single sensor system can be used to “pass” a pattern discrimination test despite providing no such capability. They further argued that the results represented the user's “recognition acuity,” maintaining that the higher than expected measured acuity demonstrated higher spatial resolution in pattern perception for the users. We previously cautioned against such mistaken interpretation.^27^ Just knowing the resolution threshold does not inform us about the visual ability with a prosthetic device. Natural vision has the benefit of a much wider dynamic range than current vision prostheses, and there is a trade-off between grayscale dynamic range and resolution, which is effectively used in halftone printing.^28^

Another example of misinterpreting the results of MAFC testing arises from a visual-to-audio SSD, the vOICe.^3^ Striem-Amit et al.^8^ tested visual acuity with this system using the tumbling E 4AFC stimuli and reported average acuity of 20/400. They label the measured resolution limit “functional resolution” and applied subsampling at the corresponding level to digital images of natural scenes. The resulting images (shown in Figure 1E of their study^8^) were of unreasonably high quality, even for views expected by patients with macular degeneration at that level of acuity. The misinterpretation is first a result of the angular dimension of acuity (20/400) being ill defined when applied to the audio SSD. In addition, the subsampled images had full dynamic range (greyscale levels), whereas the E test stimuli were binary. It has been pointed out (by Bailey et al.^24^) that “The scores from any of the various visual acuity tests should not be assumed to be measures of the person's ability to perform visually guided functional tasks in everyday life.”

## Head Scanning and Head Tracing

Another important characteristic of current prosthetic vision systems is the limited field of view, typically about 20 degrees. In retinal implant systems, this dimensionality is directly related to the retinal device size. In SSD systems, such as the BrainPort, the field of view is defined by the field of view of the camera that is transferred to the sensor, and therefore it may appear to be flexible. However, the low resolution of the electrode arrays in all systems limit the ability of the field of view to be expanded by zooming out, as that will further reduce the angular resolution of the real-world scene through the system. To utilize the limited resolution, the field of view should be limited or even magnified (zoomed-in; Jose Sahel, Saddek Mohand-Said, Paulo Stanga, Avi Caspi, Robert Greenberg, Argus II Study Group; Acuboost: Enhancing the maximum acuity of the Argus II Retinal Prosthesis System. Invest. Ophthalmol. Vis. Sci. 2013;54(15):1389). The limited field of view requires the user to scan the scene to detect and locate objects of interest. This method of use is necessary and appropriate, although very ineffective. In another variant, users are trained to scan the outline of a large or magnified test pattern contour (an action called tracing) to determine the shape of the object using head movements.^13^^,^^29^ Head tracing was frequently combined with MAFC testing and, as such, it is subject to all the limitations presented above. For example, Hallum and Dakin^14^ concluded that all eight studies of retinal prostheses that evaluated detecting the orientation of a spatial grating and applied MAFC were flawed, as they enabled head scanning (tracing) by the subjects to use integrated light perception alone (from a single sensor), as opposed to actually using spatial vision to discriminate grating orientation. There are other ways that subjects in such studies may use head scanning and tracing to masquerade as improved vision.

In a related approach, users are trained in back and forth lateral scanning of the head-mounted camera across a high contrast line on the floor, to follow the line, in what is described as a mobility task.^30^ I dubbed this mode “Radar Vision”^31^ and argued that it does not represent vision, as the same functionality may be achieved with a single-pixel sensor. Radar-like systems are only useful in largely empty or uniform space, like the sky and the ocean, with just one or a few objects of interest. In fact, such use of a prosthetic vision system is inferior to radar systems, as scanning radar not only provides the direction of the target but uses the timing of the reflected beep to provide an estimate of the distance to the target. Using the head tracing methods in training^13^ and evaluating^29^ the functionality of prosthetic vision systems needs further consideration, but a thorough discussion of this issue is beyond the scope of this paper.

## Discussion

With the three approved retinal implants not being implanted any more, it is apparent that they do not provide the expected or desired level of vision restoration. The lack of a vision-like experience is covered in much detail in a study based on systematic interviews with users.^13^ It might be that the devices failed to provide the desired effects because of technical limitations ranging from complete mechanical failure in operating (initially or over time) to insufficient resolution and extremely limited dynamic range (number of grey levels visible). Yet, they were approved by the regulatory agencies based on clinical trials that used, what this paper argues, are inadequate tests. Clearly, the implants met the testing requirements set by the companies and approved by the regulatory agencies. Many of the researchers administrating the tests in these studies insist that the implants worked well and did provide vision restoration to the blind.^11^^,^^30^ On the Second Sight web site,^32^ even at the time of this writing late in the year 2020 and after the company stopped operations, the company still claims that the Argus II devices “provide useful vision” that enables users to “distinguish and interpret light patterns” and “recognize outlines of people, basic shapes and movement.”^32^ The analysis presented here argues that despite meeting the regulatory testing requirements, the clinical trials did not actually demonstrate restoration of vision.

Hallum and Dakin,^14^ in a recent systematic review of published clinical studies of retinal implants, concluded that most studies only demonstrated improved light perception, a performance level that can be achieved with a single external sensor without implantation. Many of these studies, some that were used to gain the regulatory approval, were conducted with MAFC procedures. They also concluded that neither spatial vision nor motion perception was demonstrated with these retinal implants. In addition to problems with the MAFC in the context of the visual prostheses’ evaluations, they pointed to numerous other problems that limited the validity of those studies. These include inappropriate comparisons, lack of appropriate control (none at all, or rarely, before and after implant comparison), lack of masking of subjects and evaluators, lack of control of the placebo effect, and missing or inappropriate statistical analyses, among others. I would add to it that the intentional avoidance of full control for other sensory inputs (especially auditory) during testing,^13^^,^^30^ makes it difficult to determine the impact of the prosthesis on the MAFC performance. These issues are above and beyond the problems with the MAFC testing highlighted here.

A retinal implant that functions only as a single sensor is not justified because of the cost and the risks of invasive surgery. A retinal implant single sensor may be inferior to an external single sensor device because of the temporal characteristics of a retinal implant, such as desensitization and phosphenes persistence.^24^^,^^33^ The localization of a stimulus with retinal implant may be erroneous because of the effect of eye movements uncorrelated with a head-mounted camera, as shown by Caspi et al.^34^ Thus, although a single sensor may be a useful aid, it is not reasonable to implant devices that do not function much better than an external sensor.

Even if there is nothing wrong with an MAFC testing procedure per se, the interpretation of the results as proof of visual restoration is not supported. Many investigators design ideal observer computer programs that are able to perform an MAFC discrimination test using all the information in the image of the targets they are provided with. These programs usually perform hundreds of times better than a normally sighted observer, yet the program cannot recognize any new targets. Meanwhile, the poorly performing person has little difficulty recognizing a new target because he/she has vision. The ideal observer program would thus be useless as a robotic vision system.

MAFC testing may be used to determine the resolution limits of visual prostheses or thresholds of other relevant functions. This is an appropriate use and interpretation of the testing paradigm. By itself, however, it does not tell us much about the perception of the users. Separate evaluation procedures are needed to address such questions.^35^ It is frequently found and reported that the form of percepts, especially with multi-electrode stimulation, did not match the stimulation pattern. With the Argus II activated for the first time, it was reported, by outside observers, that “When the camera is activated and all of the electrodes can be activated together, we found participants reported that things became much more chaotic and “noisy.” The mass of flashing lights coalesces, and the vocabulary subjects use when describing their percepts focuses on changes in the overall signal (e.g. “stronger,” “more signal,” “busy,” “calm”).^13^ It is difficult to imagine how such perceptions can support vision restoration. In any case, vision is what they claim to provide, and it must be directly tested for and demonstrated.

If MAFC testing is insufficient, what might be an alternative? We have tried testing for object recognition in a single presentation, meaning that each subject sees each object only once.^36^ This may not be a practical approach. It requires either a very large number of object images or a very large number of subjects. We have found that generating an appropriate data set of such images is not a simple task, especially if one wants that data set to allow for movement and multiple points of view. The number of objects required will grow if one wants to perform repeated testing on the subjects/treated patients, and calibrating the object sets to be equivalent may not be simple.

Nevertheless, I remain optimistic that a variant of the multiple-choice testing paradigm may be developed. One possible approach was demonstrated by Striem-Amit et al.^37^ Instead of training for the discrimination of a few objects, with auditory visual SSDs, they had the subject discriminate objects taken from seven different categories. The subjects were trained on hundreds of images in each category for 70 hours. Subjects were then tested with previously unseen samples from the same categories and successfully performed the task well above chance (generalization). However, care has to be taken to ensure that the categories chosen are not discriminable by incidental low-level features. Under such conditions, the category discrimination paradigm may degenerate into a simple MAFC with the same limitations discussed above.

What is needed are testing methods that directly probe the spatial visual capabilities of the prosthetic system, demonstrating properties of vision described in the Introduction section. Teaching blind users of the vOICe soundscapes was claimed to provide the users with Gestalt perception and the ability to generalize to novel objects.^37^ Supplementary material in that paper further stated that the (congenitally) blind subjects were taught to recognize transparency, perceive the inverse problem, appreciate monocular depth cues (such as occlusion), and the variation of object size with distance. All these would constitute demonstration of vision restoration, however, no methods for these tests and no evidence were provided. Some anecdotal reports of patients’ experience may suggest vision restoration.

The ability to recognize facial expressions may be interpreted as visual experience. Stingl et al.^38^ reported that users of the Alpha IMS were able to recognize facial characteristics, such as mouth shapes (smiles) or the presence/absence of glasses. The latter is indeed a yes/no paradigm, as is the ability to distinguish light noodles versus dark beef, and red wine versus white wine. All these tasks could be performed without actually seeing the object itself. In the supplementary video,^39^ one subject reported seeing a smile after asking his friend to smile. He even insists on seeing individual teeth and the shadows between them, a performance well beyond the resolution expected or measured by the system. The same user reported not being able to see the eyes of the friend, a much larger and more contrasting detail than the shadows between the teeth. Such anecdotal reports should carry little value as evidence for vision. The ability to distinguish facial expressions was tested also with the vOICe audio system in the video supplement.^37^ In this case, it is clear that the subject is able to perform the facial expression recognition task, as it is being presented as a 3AFC distinguishing between straight line, a circle, and a third, which is none of the above. There is little doubt that the subject can distinguish the three soundscapes, but interpreting these results as an ability to distinguish facial expression is an example of a “misinterpreting the results” error.

A proper 2AFC task could be created for facial expressions using calibrated image databases of facial expressions.^40^^,^^41^ Using one such database, the experimenter would show two faces in each trial and the subject would have to say which of the two is showing a specified expression (i.e. anger). The subject should be allowed also to say: both or neither. Across trials, pairs of faces should be displayed for which each of the four possible responses is correct. The analyses of the data should follow the general directions described by García-Pérez.^42^

Recognizing facial expressions is a very high bar for a vision restoration system to achieve. Such performance is not necessary to show vision restoration and may be considered more than sufficient. However, if a prosthesis can only provide this capability, but fails to provide any of the other characteristics I listed in the Introduction section, it may not be very useful. It is important to realize, further, that if the system achieves this performance using computer neural net computation that provides the subject with the computed answer using an icon,^43^ speech, tactile, or sound, it will be a visual aid not a vision restoration system.

As in other areas of vision enhancement for the visually impaired,^44^ evaluating prosthetic vision devices may prove to be as difficult as, or more so, than designing a new prosthetic vision system. Progress in this area requires that we develop such methods.^14^ The effort required for such development is worth well beyond the regulatory need. Proper evaluation methods will support better understanding of the use and limitations of prosthetic vision and image restoration systems. In the process of developing and using such methods, we will likely uncover limitations of current systems, which we hope will guide us toward new and better designs.

Clinical tests involving one presentation and multiple possible responses are frequently referred to as MAFC tests.^24^ This term is used because MAFC tests are considered to be superior in the sense that they are free of bias. However, typical single presentation with multiple response option (MAFC) tests (such as tumbling E) are not free of bias.^24^ Multi interval test with multiple stimuli may not be free of bias either.^42^ However, neither of these aspects are at the heart of the issue discussed here. The best psychophysics testing paradigm, perfectly implemented, will not work if the task assigned to the subject does not reveal if the subject has or (re)gained vision.
